# Black, Caspian Seas and Central Asia Silk Association (BACSA) for the Future of Sericulture in Europe and Central Asia

**DOI:** 10.3390/insects13010044

**Published:** 2021-12-30

**Authors:** Panomir Tzenov, Silvia Cappellozza, Alessio Saviane

**Affiliations:** 1 Agricultural Academy, Scientific Center on Sericulture, 3000 Vratsa, Bulgaria; 2 Council for Agricultural Research and Economics, Research Center for Agriculture and Environment, Padua Seat, 35143 Padua, Italy; silvia.cappellozza@crea.gov.it (S.C.); alessio.saviane@crea.gov.it (A.S.)

**Keywords:** sericulture, agroindustry, Food and Agriculture Organization of the United Nations, mulberry, moriculture, silkworm, germplasm preservation

## Abstract

**Simple Summary:**

This paper describes the 16-years long activity of the Black Caspian Seas and Central Asia Silk Association, which was founded in 2005 to revive the sericultural activity in the area indicated by its own denomination. The reasons why this Association was established are described as a direct consequence of the decline in the sericulture agroindustry following the collapse of the Soviet Union and the world cocoon/raw silk decrease of production (except for China and India) since the 90s of the 20th century. Therefore, the enlargement of its membership to countries outside of the boundaries of the geographical area is outlined as well as its internal organization and the actions performed to promote the interaction among the member countries, especially the biyearly conferences. The international scenario is depicted to explain the criticalities experienced in promoting sericultural activities in the region, as well as the opportunities offered by the new applications of the silk, silk proteins and mulberry derivatives.

**Abstract:**

The history and recent activities of the Black Caspian Seas and Central Asia Silk Association are presented in this paper: the countries that participated in its foundation, the FAO’s action to revitalize sericulture in Eastern Europe and Central Asia, the following widening of the Association geographical limits of to enclose other European countries, which were not well-represented in other similar organizations. Some statistical data are illustrated for a better description of the scenario in which the BACSA executive board acted: the world silk production quantity and the relative production of BACSA countries in respect to the Chinese expansion. The themes treated in the BACSA conferences are reported to explain which matters the Executive Board considered the most relevant for the relaunch of this activity in relationships to the international challenges in the subsequent years; the project proposals that were presented to international donors are summarized. A SWOT (Strengths, Weaknesses, Opportunities, Threats) analysis is shown, where key-factors in determining the strengths and weaknesses of this organization and its member countries for a successful re-establishment of sericulture, are considered. In addition, future trends of sericulture with regard to innovative productions and the Green Deal are examined.

## 1. Introduction

The Central Asia region of the ex-Soviet Union includes 5 countries (Kazakhstan, Kyrgyzstan, Tajikistan, Turkmenistan, and Uzbekistan), while the South Caucasus region is composed of three (Armenia, Azerbaijan and Georgia). These 8 countries obtained their independence in 1991 after the breakdown of the Soviet Union, and since then they have experienced a dramatic economic and social crisis, resulting from the transition from a “centrally planned” economy to a “market economy”. In fact, the fast and forced industrialization of the Soviet era was full of enormous structural distortions and microeconomic ineffectiveness [1]. After the dissolution of the Soviet Union, many industrial enterprises located in this region lost their previous markets and were unable to compete under the new market conditions. As sericulture is an agroindustry, the criticalities of the Central Asian and Caucasic sericulture activities, which were experienced from the ‘90s, were thus predictable.

Similar problems occurred in the Central and Eastern Europe after 1989 (Berlin Wall’s collapse). Economies throughout the region fell into recession in the 1990s. Efforts to privatize and open markets caused unemployment and social inequality. Bulgaria’s economy contracted each year from 1989 through 1993, while Romania’s GDP (Gross Domestic Product) dropped nearly 13% in 1991 and nearly 9% in 1992 [2].

These political, social, and economic changes greatly impacted sericulture, perhaps more than any other economic activity, in the ex-communist countries. In fact, to manage the sericultural chain it is necessary to coordinate the agricultural production at the farm level, with silkworm eggs sourcing, logistic steps for drying, sorting, preservation, stocking, and marketing of cocoons; furthermore, coordination is also necessary with the subsequent steps performed in the reeling and textile plants.

What happened in the ex-communist countries in Eastern Europe and Central Asia during the transitional period from centralized to market economic system was a sudden stop of the governmental support to sericulture, the breakage of the traditional economic relationships among the countries, thus the destruction of the already established system of integration of the different processes of the sericultural production.

Until the beginning of ‘90s of the last century the approximate annual fresh cocoon production in Europe, Caucasus and Central Asia was around 50,000 tons, by this occupying the third place in the world after China and India; nearly one million farmer’s households were engaged with sericulture [3]. At the Soviet Union breakdown the Central Asian region, including Uzbekistan, Tajikistan, Turkmenistan and Iran was still a remarkable cocoon and silk producer (even now this region is engaging in the industry about 450,000 farmers) [3]; at that time the Eastern part of Europe, which was also under the communist umbrella of the USSR (Union of Soviet Socialist Republics) and also Turkey, were still producing large quantities of silk, while the Western Europe and Greek sericulture activities had already considerably declined.

Around the same period of the above-mentioned changes, i.e., in the years between 1983 and 1995, the world production of raw silk increased rather constantly and almost doubled, thanks to the exclusive contribution of China, which, in the same period tripled its production. Therefore, China in 1995 had the world monopoly of silk production with 74% of the global quantity of this fiber [4]. In the long term the constant increase of the Chinese silk that was poured over the market caused an excess in the circulating quantity of this fiber, therefore, its international price fell down (about 24% only in 1997), reaching even USD 22/kg of raw silk [4]. In this scenario only India, and Brazil, remained comparatively competitive, while all the other countries in the world were forced out of the market, or remained on the very edge (Uzbekistan, Thailand, Vietnam). This tendency to the concentration of silk production in China was even more dramatic for countries acquiring silk from Celestial Empire because of the “double price” policy, on which basis the internal silk industry could have access to raw material at a price 20% lower than that for foreign buyers [4]. The monopoly of silk production, joint to the double price policy, permitted China to obtain the absolute leadership in the cost of raw silk and even of silk tissues and clothes [4].

## 2. Establishment and Purpose of the Black and Caspian Seas and Central Asia Silk Association (BACSA)

As mentioned above sericulture is an agro-based industry and agriculture has always been of huge importance for the countries of the Black and Caspian Seas and Central Asia area, supporting the income and providing employment to a large majority of inhabitants of the rural territories, but also protecting the environment through the sustainable use of natural resources. For this reason, the collapse of sericulture following the USSR dissolution was regarded with much concern by NGOs (Non-Governmental Organizations). The case of Uzbekistan is emblematic: due to the combined effect of the economic turmoil after the independence, and the Chinese monopoly of silk prices, Uzbek cocoon production dropped from 33,000 t in 1990 to 21,000 t in 1997 [5].

In order to counteract this decline in sericulture for the countries of the former USSR or previously enclosed under its political umbrella, in April 2005, the Food and Agriculture Organization (FAO) of the United Nations and the Uzbek Government organized in Tashkent, Uzbekistan, the “International Workshop on Revival and Promotion of Sericultural Industries and Small Enterprise Development in the Black, Caspian Seas and Central Asia Region”. Twelve countries including Azerbaijan, Bulgaria, Egypt, Georgia, Greece, Japan, Kazakhstan, Republic of Korea, Tajikistan, Turkey, Ukraine, and Uzbekistan participated in this meeting. During the workshop a regional association, named Black, Caspian Seas and Central Asia Silk Association (BACSA) was established [5].

The main targets of this association were to generate sericulture projects from external resources, including bilateral and multilateral cooperation, sensitize respective governments and prospective donors, promote local and regional joint efforts, which would have allowed the cooperation among the countries of the region of Europe, Caucasus and Central Asia, develop concrete actions to fortify the sustainable development of the sericulture in the region, promote agreements for international scientific-technical cooperation and business relations among the countries involved, encourage market studies, training, and spreading sericultural germplasm, and silkworm eggs. In fact, the BACSA member countries have many problems and issues in their sericulture revival and development, which could be partly or completely solved by the help of regional cooperation among them. Such problems are the access to the EU (European Union) funds for research, specializations and training of students and technical personnel, exchange of sericulture germplasm resources and improvement of the mulberry sapling and silkworm egg quality; other issues consist of saving the sericulture germplasm of the BACSA region, increasing silkworm egg production or favoring its revival in some of the member countries, solving criticalities of cocoon and silk marketing, improving the raw silk quality and rising the share of exported or locally processed raw silk, producing small niche textiles from cocoons and developing silk handcrafts [6]. 

At the time of its establishment, BACSA member states that were producing silk carpets, such as Iran and Turkey, were also comparatively big raw silk importers as the local silk production was not able to satisfy the silk carpet needs of raw material [7]. In that period, as even presently, some BACSA countries did not have any own silkworm egg production and in some of the Central Asian countries the quality of currently locally produced silkworm eggs did not meet the international standards, or their quantity could not satisfy the local needs [8]. 

Therefore, one of the basic aims of expansion of BACSA inter—regional cooperation was/is to transmit and share sericulture germplasms, silkworm eggs, advanced technologies, training, dry cocoons, raw silk, and silk allied products.

## 3. The BACSA Activity

### 3.1. The BACSA Composition

When established in 2005 BACSA included 9 countries—Azerbaijan, Bulgaria, Georgia, Greece, Kazakhstan, Tajikistan, Turkey, Ukraine and Uzbekistan. Later on, and gradually, BACSA attracted for membership new countries—Albania, Armenia, Iran, Poland, Romania, Switzerland, Italy, Spain, Germany, Portugal, Slovenia, Russia, UK so that currently the association includes 22 countries and has also 64 individual members and 4 institutional members. The individual members from countries, which are completely out of the enlarged BACSA region are mostly from India (43), but also from China (2), Egypt (2), Korea (1), Ghana (1), Syria (1) and Indonesia (1). Presently BACSA has 26 members of the Executive committee from 22 different countries. The composition of the Executive committee has been totally renewed at the end of 2009 [9] and can be periodically revised according to changes in the membership.

### 3.2. The BACSA Structure

The management of the association is composed of a President, two Vice-Presidents, one national coordinator for each member country, which are the most representative part of the Executive Committee; all of them are democratically elected. The members of the Executive Committee are the direct coordinators of all the current activities for each country, within the regional context. The Executive Committee is the bridge among each country, the national coordinator and the other countries of the association, to execute the actions defined in the region. The Committee gathers in person at least once every two years; its members maintain contacts among themselves regularly by e-mail/phone, to perform the following functions [9]:

- To evaluate the work made by each national coordinator in the respective countries in relationship to BACSA.
- To recommend people in the association who should receive training abroad.
- To evaluate and to watch the handling of the “Rotatory Funds” and “Research Funds” possibly created and to give recommendations on the best use of these resources.
- To present/display the research proposals that require financing from the “Research Fund” and to approve the necessary resources for this aim.
- To give recommendations and suggestions on all publications and written material that are created within the framework of the BACSA.
- To advise the association’s President on the advances and progresses that take place in the development of the activities and give recommendations to her/him on possible modifications and corrections.
- To decide the main BACSA activities, including accepting new members etc.

### 3.3. The BACSA Conferences

Until the end of 2019 BACSA organized 9 international conferences. Each conference was on a specific subject, connected with the problems of regional sericulture development. The BACSA conferences are shown in Table 1.

The main findings, conclusions and recommendations of these international meetings are listed below:

(1) Revival and Promotion of Sericultural Industries and Small Enterprise Development (2005): As recalled before, this conference posed the basis for the development of a short/medium-term strategy for the sericulture revival at a regional level and preparation of projects for the sericultural industry rehabilitation in the region countries, ready for donor search [6].
(2) Silk Handicrafts Cottage Industries and Silk Enterprises Development (2006): This conference was particularly focused on the creation of a network collaborating in the different sectors of the supply chain to improve competences in silk reeling and silk industry management specially to establish ecofriendly processes of dyeing and finishing and to promote silk handcraft production and export to foreign countries even through the touristic channel [7].
(3) Sericulture Challenges in the 21st Century (2007): The most important theme of this meeting was the preservation and exploitation of germplasm resources (silkworm and mulberry) of the region, which are particularly rich but at risk as their preservation is costly for the concerned countries [7].
(4) Possibilities for Using Silkworm and Mulberry for Non-Textile Purposes (2008): The target of the discussion was the proper utilization of secondary and waste products of the sericultural industry, which can generate an extra income in addition to silk, which represents the main output. The by-products of sericulture are sericin, pupae, moths, silkworm frasses, silk waste, mulberry branches, fruits and roots. New commercial products can be obtained from these raw materials with a valuable destination for pharmaceuticals, cosmetics, feed and food, new materials [10].
(5) Sericulture for multi products—new prospects for development (2011): This conference enlarged the issues treated in the previous one of 2008, particularly stressing the criticalities of non-textile silk production, from the scientific and regulatory point of view [10,11,12].
(6) Building Value Chains in Sericulture (2013): Stakeholders of different BACSA countries met to plan possible cooperation programmes in the field of science and technology transfer, education and training with the aim of promoting bilateral research projects, financed by each participating country’s government through grant competitions, announced periodically by the Ministries of education and sciences or bi and/or multilateral projects, financed by the EU, in addition to specialization and training of students and technical personnel in leading research centers and commercial companies, financed by EU/national programmes [13].
(7) Organic Sericulture—Now and the Future (2015): The theme of this meeting was about the new trend of organic production in agriculture that well adapts to sericulture, especially in Europe, where mulberry cultivation has always been “naturally organic”. How to develop a certification chain for textile and non-textile organic silk production and how to exploit this certification on the market were the key-points treated in the internal discussion [14].
(8) Climate changes and chemicals—the new sericulture challenges (2017): In most of the 22 countries currently members of the Black, Caspian Seas and Central Asia Silk Association, wherever they are, Europe, Caucasus and Central Asia, sericulture has been very negatively affected by the use of chemicals in agriculture and by climatic changes. In fact, through fluctuations in temperature, water regimes and the increase in carbon dioxide levels, global climate changes directly influence mulberry, soil, pests, and silkworm rearing. The specific climatic conditions in the BACSA region countries require mulberry to have high cold and drought tolerance and the silkworm strains to possess a good tolerance to adverse rearing conditions like high temperature, daily temperature fluctuations and feeding with coarse mulberry leaves. On the other hand, the wide use of insecticides can easily harm silkworm rearing and even completely destroy the whole sericulture value chains in some regions or countries. A common effort to develop new strategies is required to mitigate the joint effects of the chemical use and climatic changes [15].
(9) Sericulture preservation and revival—problems and prospects (2019): During this conference the BACSA members discussed about the world trends of silk production, in particular the decrease of the cocoon quantity annually available in China and the increase of the international price. These phenomena give a prospective of decrease in the silk use by the textile industry and in coming back to consider this fiber as a very luxury good for a niche market. Sericulture may change from an industry for the poorest farmers, to an agribusiness, requiring more investments and production costs, but having high revenues because of the high market price of the products. BACSA countries should be prepared for that [3,16,17].

The BACSA conference foreseen for 2021 was postponed because of the COVID 19 problems.

### 3.4. The BACSA Project Proposals 

The BACSA activity to prepare project proposals is illustrated in Table 2. This activity was very intense between 2006 and 2010, then after many failures, these kinds of attempts ceased in terms of projects studied for the whole area and focused mostly on more limited projects or bilateral agreements between members of BACSA [18]. 

### 3.5. The BACSA Network for Facilitating Commercial Contacts among Stakeholders

BACSA makes all the efforts to establish connections among the producers, sellers and buyers of different sericultural products such as mulberry saplings, silkworm eggs, dry cocoons, raw silk, silk yarn, fabrics, and garments. These activities are performed through regular updating of the section “sell/buy information” on the BACSA website as well as responding in real time to all the enquiries from possible sellers and buyers, connecting stakeholders together and giving a chance to exhibit sericultural products by organizing international workshops, conferences, etc. [19]. 

### 3.6. Results of BACSA Global Activity

BACSA carried out a series of activities that can be better understood when we compare the situation before 2005, when BACSA was established, and at the present time; from this comparison it is evident this association’s role in the regional sericulture preservation, revival and development (Table 3).

## 4. BACSA Becomes Attractive for Western and Central Europe Countries

As mentioned before, the situation of sericulture of the countries of Western and Central Europe non-belonging to the Communist block was even worse than that of BACSA countries, because here the decline of sericulture (referred to as agricultural and reeling activity) began much earlier than in the ‘90s of the last century and precisely after the second World War with the maximum peak between the ‘60s and ‘70s. On the other hand, these countries retained a flourishing silk industry (weaving, dyeing, printing, manufacturing), especially in Italy, France, Switzerland and England [9]. The main feature of this silk industry has been its complete dependence for silk from external sourcing, especially from China, for a long time [9]. During the ‘90s the international price of silk was low and the quantity on the market abundant; this was the reason why the European silk industry was not stimulated to look for other sources than China. However, some signals of market sufferance were already very clear: double pricing of silk and difficulty in finding a constant and high quality of this fiber production in China [4]. 

In Western Europe some residual cocoon production could be preserved due to the EC (European Communities) subsidies to sericulture (Regulation EEC n. 922/72) establishing a contribution per each reared silkworm box from which a minimum quantity of at least 20 kg of fresh cocoons was obtained. This contribution was completely stopped in Europe in 2014, due to the Common Agricultural Policy (CAP) reform; the EC governments were allowed to support some strategic sectors only with direct payments; the majority of EC countries decided that sericulture was not a priority, while only the government of Greece continued to pay subsidies to its farmers for fresh cocoon production.

In Italy and France this lack of support from the Government was partly justified by the catastrophic situation of sericulture caused by external factors; in fact, from 1989 to 2010 France and Northern Italy (where some cocoon production had been preserved even in a small amount) were affected for a long time by the pollution due to an Insect Growth Regulator (active ingredient: fenoxycarb, commercial name: Insegar). The chemical was sprayed on the fruit orchards and its drift was transported by the winds onto mulberry leaf. It was so effective to be capable of acting on the silkworm larvae at doses of nanograms (1.0 × 10^−9^ g) or even lower [10]. Due to this phenomenon the larvae were unable to spin their cocoons (“non-spinning syndrome”) [11,12] and French and Italian rearers were completely discouraged from continuing to practice sericulture. For almost 20 years, until the date of the retirement (which was operated by the same producing company) of the chemical from the market and exhaustion of the stocks, it was impossible to rear silkworms in the above-mentioned areas.

In 2009 the INRA (National Research Institute for Agriculture) Sericultural National Unit of Lyon (France) closed and only the Sericulture Laboratory of CREA (Council for Agricultural Research and Economics) in Padua remained responsible for the preservation of all the genetic resources of Western Europe [13]. Furthermore, the International Sericultural Commission based in France, transferred its headquarters to India, which was a signal of disengagement of the European stakeholders from sericulture [14]. 

In the same period the international price of raw silk began to rise very rapidly, reaching levels of about USD 55–60/kg mostly because of the industrialization of China in the last decades, in particular the Eastern area of the country, which was traditionally more apt to sericulture. Although the Chinese government made a big effort to transfer sericulture to other regions, less industrialized and more devoted to agriculture, the silk quantity and mostly its quality decreased significantly; as a consequence, the price of the best quality of silk in the international market began to increase quite abruptly. The COVID 19 pandemic has been a brief parenthesis in this trend.

Therefore, during the last 10 years a new interest was expressed by the European silk industry (especially from Italy and Switzerland) for countries alternative to China where silk might be produced; on this basis the silk industry may re-consider establishing part of the cocoon production they need in Europe, Caucasus and/or Central Asia. A sign of this interest was the BACSA conference of 2013, which was held in Italy with the economic support and commitment of the Italian silk industry in collaboration with CREA (the Research organ of the Italian Ministry of Agricultural, Food and Forestry Policies) [15]. A delegation of the executive board was hosted in Como and visited “Ratti”, one of the most important silk Italian companies belonging to the Marzotto group. Another sign of interest was the progressive association to BACSA of countries from Western Europe: Italy, Germany, Spain, Portugal, Slovenia, Switzerland, UK which are currently members of BACSA [16].

However, the restoration of the sericulture chain in Europe and BACSA countries is a very large goal that cannot be sustained by the industry alone; a public-private partnership should be envisaged, and the governmental and EC support should be provided to make it a realistic goal, which, however, needs long term investments.

## 5. Problems and Prospects

**Figure 1:**
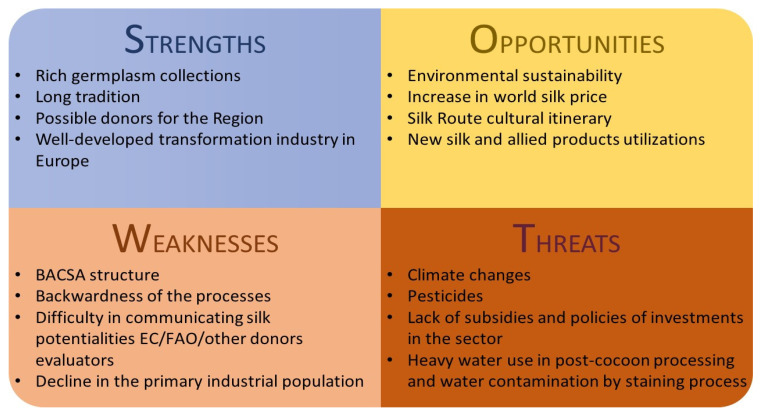
SWOT analysis representing criticalities and qualities of BACSA examined with respect to the possibilities of success in revitalizing the sericultural activity in the area.

In this section BACSA will be examined according to the problems and prospects that characterize this organization. An attempt to summarize the criticalities and “plus values” of BACSA is reported in Figure 1.

### 5.1. Problems Related to the BACSA Structure

BACSA is a quite open and very flexible organization; the membership of the Black, Caspian Seas and Central Asia Silk Association (BACSA) is possible for all individuals/institutes/countries from Europe and Central Asia which are willing to share common goals for an industrial and economic growth through sericultural activities. The expected members may be of two categories: “Internal” from the same geographical region countries, and “External” from other countries, who are possibly interested in technical cooperation or business with Europe and Central Asia region as well as from those countries that are possible donors for sericulture revival projects [20]. Even Chinese, Indian, Japanese, Korean, African scientists/stakeholders applied to this organization for membership. Chinese and Indian delegations are admitted as observers to BACSA meetings. No membership fee is required so far, and this fact permits to participate even to poor countries or individuals. However, what in principle appears to be very democratic and egalitarian might be a problem in the future in case some divisive themes are discussed, or the interest of some single country is concerned. At the moment, the BACSA community reaches a good agreement on any decision basically because of three factors: (1) members are few and many of them have been known each other for years; (2) there are no huge interests at stake because sericulture in the BACSA countries is mostly confined to a niche economy; (3) China’s presence in the silk world is so outstanding that any other country should decide its strategy to develop its own silk production/industry reacting to the Chinese monopoly more than fighting against other competitors. Therefore, it is possible that this association should assume another structure in the future; in fact, some problems are the same for most of the countries, others are typical of a more restricted geographical area. The three macro-areas that can be outlined in the BACSA region are Central Asia, the Caucasus and Europe. It might be advisable in the next period to divide BACSA in three different working groups, joining together only on themes of common interest. For example, the income level of farmers in these three macro-areas is completely different, being the minimum in Central Asia, maximum in Western Europe. It appears very clear that Europe should make a big effort to mechanize and specialize its silk production, while countries like Uzbekistan (Central Asia) have the potential of producing high quantity of cocoons at a comparatively low price by exploiting local manpower and can be identified as competitor with China in the middle term. Other principles to form working groups may be assumed: for example, silk industrial converters versus cocoon producing/reeling countries, low quantity silk producing countries versus high quantity silk producing countries, etc.

### 5.2. Common Problems

#### 5.2.1. Preservation of Genetic Resources 

**Figure 2:**
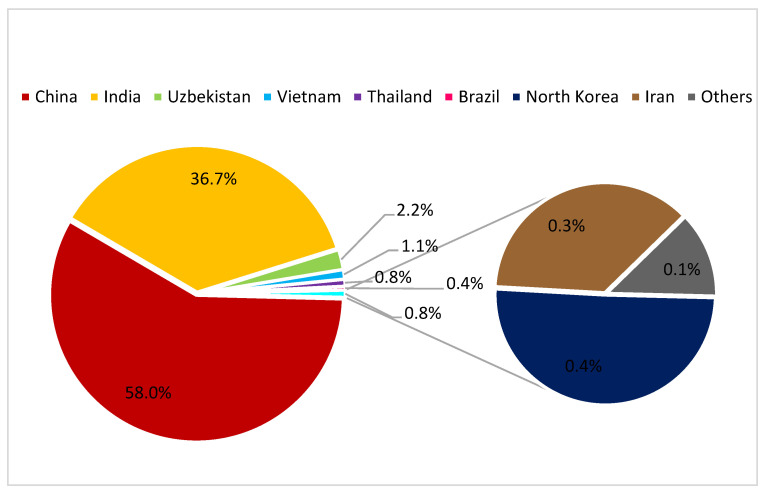
Pie-chart and relative shares of global silk production (data from https://inserco.org/en/statistics; accessed on 23 November 2021) in 2020; China and India alone cover more than 90% of the worldwide production (94.7%).

The global raw silk production was around 91,945 t in 2020, but out of them 53,359 t were produced by China and 33,770 t by India, while all the other countries produced only about 4816 t of raw silk [21] (see Figure 2). 

That means almost 95% of the total world silk production is from only two countries—China and India. On the other hand, although many countries in the World dealt with sericulture in the past or are still dealing nowadays, the majority of them only make efforts to preserve this activity and only few of them to revive the sericultural industry. Silk production volumes more than doubled from 1990 to 2019 but it saw a decrease over the last five years. Even in those countries that are the biggest world cocoon and silk producers, there are presently entire regions where the sericultural activities have been partly or even completely stopped and the sericultural expertise may be lost. Among these, BACSA associated countries, which still represents the third world producing area, face problems typical of nations with history of long tradition and scarce current production. The first problem for them is how to preserve their mulberry and silkworm germplasms, which usually constitute public genetic resources, mostly located in research institutes [8,22]; the preservation activity is quite expensive, governments, in consideration of the present scarce economical revenues from the sericultural agribusiness, tend to restrict funds destined for conservation. Any hypothesis to concentrate the germplasm in one center only, preserving accessions for all the region, is quite unrealistic and in addition dangerous for the possible losses of genetic material in case of diseases or unforeseen accidents. The best way to preserve genetic resources is their spreading ex-situ; however, this proved to be very difficult because of intellectual properties on the selected strains and varieties and could be done only for the genetic material that does not have any economic significance. Therefore, encouraging the use of these resources and restarting an economically viable sericulture is certainly the best way to guarantee their preservation. A contemporaneous rehabilitation of the activity in the whole BACSA region does not appear to be necessary; a partial restarting in those countries still endowed with well-preserved genetic material might also be useful for those countries that have lost their own sourcing, due to different accidental events, or have germplasm of inferior quality; in fact, even a partial sericultural revival will favor bilateral exchanges and commercial exploitation agreements among different countries, which might be regulated internally within the BACSA framework.

#### 5.2.2. Backwardness of the Agroindustry Chain

Another problem for BACSA countries is the backwardness of the agricultural process (mulberry cultivation and silkworm rearing) and/or the silk industrial transformation. This is partially because the Chinese monopoly on silk has maintained too low the silk price to stimulate innovation and mechanization in countries with capitalistic economies; the only advanced part of the silk chain, in these countries, regards industrial processing after reeling, in which modernization and digitalization play a basic role on the final quality of manufacts for the exclusive fashion industry of the most important world brands. The communist governments highly promoted the mechanization and technical advances in the agriculture. In the communist countries after the ‘60s of the 20th century a big industrialization occurred; thus, moving of a huge mass of people from the rural areas to the cities resulted in the lack of agricultural workers. For this reason, in the ‘80s the mechanization in agriculture was a priority. Even in sericulture, where cocoon production, in some communist countries, was in the hands of the big agricultural cooperatives or state enterprises, they managed to organize large scale silkworm rearing, mechanized feeding of young instar larvae, using Japanese machineries, cardboard frame mountages, machines for cocoon deflossing. However, most of these efforts in sericulture appeared to be not economically effective due to the Chinese concurrence and were abandoned. To recover the technological gap of sericulture with respect to other agricultural crops or agro-industrial chains, more competitive in terms of economic revenues for farmers or investors, is not easy and requires many funds to develop innovations. This is one of the main reasons why sericulture is a niche production in several developed countries. This technological gap in sericulture became more evident in the last decades when the effect of climatic changes and environmental pollution began to seriously affect agriculture. For example, dramatic climatic changes began to negatively affect silkworm rearing with serious fluctuations in the average temperatures even in the seasons traditionally favorable to sericulture; the lack of climatic control of silkworm rearing facilities and of digital automatic control of temperature and humidity can cause big problems to larval development; heavy droughts, late frosts, excessive rains compromise mulberry leaf harvests; high temperatures and humidity favor spreading of insect pests, which are also more invasive due to the increased globalization of the transport of goods around the world, which works as an involuntary carrier. Fighting against these new insect pests with insecticides, in turn, affects sericulture. To solve these problems a lot of technology and research related to the environmental management of sericulture would be necessary; however, as previously mentioned, available funds to improve knowledge in the sericultural field are very limited [16]. 

#### 5.2.3. Funding Access 

With regard to structural funding for research, training, dissemination, demonstration and other activities related to the sericulture revival, as mentioned above, some BACSA associated countries are members of the EU; therefore, they should have access to EU funding for research institution and SMEs (Small and Medium Enterprises). However, until now the joint attempts for funding common projects have generally failed [18]. It is likely EC evaluators think that silk-focused projects have a minor impact on EC country development, with other agricultural/industrial activities considered more important than sericulture. Probably, the correct manner to attract financing is to enclose sericulture as a small part of wide projects focusing on other activities and where sericulture represents a case-study more than the central research or investment attractor. On the other hand, non-EC BACSA countries can have access to FAO funds or to those of other NGOs [18]. However, the lack of experts at the world level in the specific branch of sericulture often results in a minor attention to this theme. For example, two Korean experts gave a great personal contribution (Dr. Hoo Zoo Lea, FAO Senior officer and Dr. Jong Sung Lim, FAO consultant) to the BACSA creation, because of their knowledge and in-depth expertise in this sector [6]. Unfortunately, FAO now is missing these professional officers specialized in sericulture.

#### 5.2.4. Governmental Subsidies

With regard to subsidies of governments to silk production, what occurred was that both in the EC and in the other BACSA countries, where they were applied, they did not prevent sericulture from the decline and, in some cases, they triggered fraud from farmer associations or other organizations. Therefore, it is clear enough that this instrument to guide the market might be useful only if coupled with a general policy of industrial and technological development of this sector.

### 5.3. Prospects for the Future

China, India, Brazil have probably already reached their maximum level of silk production and are not going to increase further their quote in the world market [23]. On the other hand, silk consumption, so far, has been a very small quote of total world fiber production (less than 1%) [24], but very stable over time, although with a diminishing trend in the last 5 years [21]. This stability of silk for the textile market is due to the general buyers’ identification of silk with a luxury fiber, which is a part of human civilization; in many countries it is intrinsically connected with local culture, and it has a long-standing tradition. Silk is considered as “Queen of textiles”; it has some unique and important characteristics, such as its ability to keep the body warm when it is cool, and cool when it is warm, or being a healthy fiber because it breathes easily and naturally keeps away moisture from the skin or being actually soothing to skin diseases and itches. Therefore, according to the recent trend for a rising demand of natural fibers from the final consumers, who look for comfortable wearing and sustainability of clothes, an increase of the silk price is expected; this phenomenon might give opportunities to BACSA countries to be competitive with China, even considering that the top world quality silk fabrics and garment producing industries are concentrated in Europe (Italy, France, Switzerland, England). 

Many BACSA countries might expand their artisanal and handicrafts production, especially by linking it to the tradition of their territories, countryside landscapes, culture. This idea gave birth to a project promoted by the Venice Municipality and the Council of Europe through the creation of a cultural itinerary “The European Silk Route”; it aims to be a local cultural network and infrastructure linking cities, regions, sites, museums and universities in order to enhance knowledge of a shared European cultural heritage, both tangible and intangible, and to promote new relationships within Europe and between Europe and the East through sharing of best practices and cultural tourism activities. The route would ideally be based on Marco Polo’s travels eastward and include silk production and trade itineraries in Europe in the following centuries. The narrative to be developed will start from the commercial and religious exchanges that took place along the silk road, and specifically Marco Polo’s trips. It will then analyze silk’s impact in Europe through four main themes: the textile activity (from artisanal production to industrialization: innovations, technologies, the work world); silkworm rearing and its social, economic, agricultural and environmental consequences; the use of silk in paintings, fashion and design; and research and development in silk production [25]. The work undertaken to reach the certification of the cultural itinerary from the Institute of Luxembourg began in 2018 [25].

In addition to this exciting opportunity, the forecasts for the future represent that the demand of non-textile silk as constitutive proteins will increase at a steady rate, due to the new utilizations of silk as a versatile polymer for different aims (cosmetics, pharmaceuticals, biomedicals). Recently bio-technological sericulture has been developing [26,27]. For the first time in the world, in 2017, the legitimated rearing of genetically modified silkworms in conventional sericulture farms started in Japan. Functional silk is a promising material for medical applications. Using the methods of genetic engineering, absolutely new silks that have unprecedented functions were developed. These are transgenic spider silk [28], hyperfine silk of small diameter, artificial blood vessels, fluorescent silk [29]. Some of the BACSA countries are ready to face this biotechnological challenge. Silk regenerative medical materials like silk sponge, silk hernia mesh, wound dressing, silk surgical tape, hydrogel, films and 3D scaffolds for wound healing and tissue regeneration and reconstruction gels, powders, enzyme immobilization matrices were also created [30,31,32,33,34]. Transgenic sericin is used for several medical reagents, like blood test drugs, biomatrix for tissue engineering and cosmetics [35,36,37,38]. 

Therefore, new kinds of applications are likely to offer new opportunities; the interesting consideration is that, in this case, it is not necessary to produce silk in a huge quantity and it is not necessary to possess big reeling plants or transformation industries. These opportunities might allow BACSA countries to increase their production slowly and steadily. The region possesses some of the richest silkworm and mulberry germplasm collections. Several of the commercial silkworm hybrids, produced in the BACSA area manifest comparatively high productivity, namely single cocoon weight 2.2–2.5 g, shell ratio 23–24%, shell weight 0.500–0.600 g, filament length 1300–1500 m under laboratory conditions [39,40]. This might help in making the member countries attractive for this production. The level of sericultural science and technology in the region countries is comparatively high at a world level. This expertise might be particularly useful because new technological properties might be required for such a kind of silk production for innovative aims.

The EC green deal might also play a great role in promoting the development of sericulture in the BACSA countries: as mentioned before, sericulture might be an organic agricultural practice; the mulberry is environmentally useful to protect soil from erosion, to adsorb carbon dioxide, to prevent desertification in marginal areas; if it is exploited in a non-intensive way it requires limited fertilization and irrigation and no pesticides at all; moriculture can also be practiced in polluted or salty soils to accelerate their recovery to production [41,42,43]. The silkworm is an environmental sentinel especially informative on the abuse of pesticides on agricultural crops cultivated nearby the rearing places. Furthermore, sericulture and moriculture can be exploited for circular economies where by-products of some processes can become raw materials for others. Mulberry fruit can be consumed fresh, dry or employed for production of juice, wine, jam and food additives. Pharmaceuticals can be extracted from mulberry branches, roots, leaves (for example, 1-deoxynojirimycin (DNJ) with antidiabetic aims [44]).

## 6. Conclusions

BACSA, being established only 16 years ago, is a rather young international organization, for example in comparison to another one in the same sector, the International Sericultural Commission (1960). BACSA is basically managed on a voluntary basis thanks to the work and support of individual members, mostly belonging to scientific institutions. According to its aims, it has been strengthening links and sharing knowledge among sericultural member countries, by giving a wide support to many actions dedicated to the revival of sericulture in Europe, Caucasus and Central Asia. Although this revival has not been possible yet on a large scale there are many hints about possible future developments, so that the support action of this Association continues to be fundamental and would deserve more attention by the sector stakeholders. 

## Figures and Tables

**Table 1:** BACSA conferences (2005–2021).

BACSA Conference Title	Year	Location
(1) Revival and Promotion of Sericultural Industries and Small Enterprise Development	2005	Taskent, Uzbekistan
(2) Silk Handicrafts Cottage Industries and Silk Enterprises Development	2006	Bursa, Turkey
(3) Sericulture Challenges in the 21st Century	2007	Vratsa, Bulgaria
(4) Possibilities for Using Silkworm and Mulberry for Non-Textile Purposes (First Balkan workshop)	2008	Plovdiv, Bulgaria
(5) Sericulture for multi products—new prospects for development	2011	Bucharest, Romania
(6) Building Value Chains in Sericulture	2013	Padova, Italy
(7) Organic Sericulture—Now and the Future	2015	Sinaia, Romania
(8) Climate changes and chemicals—the new sericulture challenges	2017	Sheki, Azerbaijan
(9) Sericulture preservation and revival—problems and prospects	2019	Batumi, Georgia

**Table 2:** BACSA project proposals to different donors (2006–2021).

Presented Projects	Year of Proposal or Realization	Possible Donor	Financed (Yes/No)
Improvement of Income-Generation Options Based on Revival of Sericultural Industries and Promotion of Small Silk Enterprise Development in Eastern Europe and Central Asia—Concept Note	2006	UNDP, ADA, CIDA, DCI, DEZA, DFID, FAO, GTZ, IFAD, ITC, UNCTAD, JICA, KOICA, NORAD, SIDA, UNIDO, USAID, EBRD, World bank	N
Comparative studies of silkworm hybrids performance for sericultural enterprise development in Black, Caspian seas and Central Asia region	2006–2007	FAO	Y (only partially)
Support for unlocking and developing the research potential of silkworm breeding and innovative management techniques in Bulgaria, Greece and Romania, targeting to the small silk enterprise development	2007	EU FP 7 “Capacities” Program	N
Improvement of Income-Generation Options Based on Revival of Sericultural Industries and Promotion of Small Silk Enterprise Development in Eastern Europe and Central Asia—Full Proposal	2008	FAO	N
Regional sericulture germplasm resources network for Africa, Middle East, Central Asia and Europe	2008	FAO	N
Proposal of a Regional workshop on “Utilization of mulberry and silkworm genetic resources for sericultural enterprises development in Africa, Middle East, Central Asia and Europe”.	2008	FAO	N
TCP/ALB 3101 “Revival and Development of Sericulture in Albania”	2008–2009	FAO	Y
SERINNOV– SEE EoI/B/481/1.3/X “Sericulture and silk products in South-East Europe—stepping from the tradition to innovation and strengthening the economic profile of respective regions	2009	EU—SEE program	N
Exploitation of the heavy metal movement and removal pathways and mechanisms in the mulberry– silkworm chain for the establishment of model applications for further practical use in bioremediation of contaminated soils.	2009	FP7—KBBE program	N
TCP/GEO/3201 “Sericulture sector study in Georgia”	2009–2010	FAO	Y

**Table 3:** BACSA’s activity results (2005–2021).

Item	Situation in 2005	Situation in 2021
Contacts among the key specialists and institutions engaged in the sericultural industries	Very few, well-developed among some of the Ex-Soviet Union and Eastern Europe countries only	Good contacts among all BACSA member countries, key sericulture specialists and most of the institutions
Regional sericulture database	Not available	Available, uploaded on the BACSA website and regularly updated
Number of countries, associated in BACSA	Only 8 Eastern Europe, Caucasus and Central Asia countries	22 countries from Europe, Caucasus and Central Asia; out of them 9 EU member states, which give possibilities to apply for projects financing from the EU funds.
Enquiry system for sericultural products marketing	Not available	Available: enquiries to BACSA → distribution of the enquiries to the national coordinators → distribution of the enquiries to interested stakeholders in each member country for direct contacts; uploading the sell/buy enquiries on the BACSA web site.
Exchange of sericulture germplasm resources among the BACSA member countries	Very few, well-developed among some of the Ex-Soviet Union and Eastern Europe countries only	At a much higher scale, based on bilateral scientific projects
Export of mulberry saplings and silkworm eggs	Very few, among some of the Ex-Soviet Union states only	There is, but still in a too small scale
Regional conferences and meetings	Very few, well-developed among some of the Ex-Soviet Union and Eastern Europe countries only	Regular BACSA international conferences, at intervals of 2 years, 9 conferences already organized.
Specializations and training of students and technical personnel in leading research centers and commercial companies in BACSA member countries	No	Present, but still in a too small scale.
Sensitizing the national governments about the regional BACSA executive meetings results, decisions and follow ups	No	Yes, operated by the BACSA national coordinators
Responding to different enquiries, concerning the sericultural industries in the region	No	Yes, operated by BACSA president and national coordinators
Supporting the sericultural institutions and specialists in the BACSA member countries by providing, when necessary, letters of support, personal recommendations, reviews of scientific monographs and doctoral dissertations etc.	No	Yes, operated by BACSA president and selected experts
Popularization of Europe and Central Asia sericultural industries in the world through participation to international conferences and meetings	Scarce	Yes
Development of regional sericulture projects and looking for donors	No	Yes
Promoting bilateral and multilateral agreements for cooperation among the BACSA member states	No	Yes

## Data Availability

Data summarized in Figure 2 are available on the INSERCO web site at the following section/address: https://inserco.org/en/statistics (accessed on 23 November 2021).
